# The Netherlands Is on Track to Meet the World Health Organization Hepatitis C Elimination Targets by 2030

**DOI:** 10.3390/jcm10194562

**Published:** 2021-09-30

**Authors:** Marleen van Dijk, Sylvia M. Brakenhoff, Cas J. Isfordink, Wei-Han Cheng, Hans Blokzijl, Greet Boland, Anthonius S. M. Dofferhoff, Bart van Hoek, Cees van Nieuwkoop, Milan J. Sonneveld, Marc van der Valk, Joost P. H. Drenth, Robert J. de Knegt

**Affiliations:** 1 Department of Gastroenterology and Hepatology, Radboud University Medical Centre, 6525 GA Nijmegen, The Netherlands; joostphdrenth@cs.com; 2 Department of Gastroenterology and Hepatology, Erasmus University Medical Centre, 3015 GD Rotterdam, The Netherlands; s.brakenhoff@erasmusmc.nl (S.M.B.); m.j.sonneveld@erasmusmc.nl (M.J.S.); r.deknegt@erasmusmc.nl (R.J.d.K.); 3 Department of Gastroenterology and Hepatology, University Medical Centre Utrecht, 3584 CX Utrecht, The Netherlands; c.j.isfordink-3@umcutrecht.nl; 4 Division of Infectious Diseases, Department of Internal Medicine, Amsterdam Infection & Immunity Institute, Amsterdam UMC, University of Amsterdam, 1105 AZ Amsterdam, The Netherlands; m.vandervalk@amsterdamumc.nl; 5 Health Economics and Outcomes Research, AbbVie Inc., North Chicago, IL 60064, USA; wei-han.cheng@abbvie.com; 6 Department of Gastroenterology and Hepatology, University Medical Centre Groningen, 9713 GZ Groningen, The Netherlands; h.blokzijl@umcg.nl; 7 Department of Medical Microbiology, University Medical Centre Utrecht, 3584 CX Utrecht, The Netherlands; g.j.boland@umcutrecht.nl; 8 Department of Internal Medicine, Canisius Wilhelmina Hospital, Radboud University Medical Centre, 6532 SZ Nijmegen, The Netherlands; a.dofferhoff@cwz.nl; 9 Department of Gastroenterology and Hepatology, Leiden University Medical Centre, 2333 ZA Leiden, The Netherlands; b.van_hoek@lumc.nl; 10 Department of Internal Medicine, Haga Teaching Hospital, 2545 AA The Hague, The Netherlands; c.vannieuwkoop@hagaziekenhuis.nl

**Keywords:** hepatitis C, HCV, elimination, model, COVID-19

## Abstract

Background: The Netherlands strives for hepatitis C virus (HCV) elimination, in accordance with the World Health Organization targets. An accurate estimate when HCV elimination will be reached is elusive. We have embarked on a nationwide HCV elimination project (CELINE) that allowed us to harvest detailed data on the Dutch HCV epidemic. This study aims to provide a well-supported timeline towards HCV elimination in The Netherlands. Methods: A previously published Markov model was used, adopting published data and unpublished CELINE project data. Two main scenarios were devised. In the Status Quo scenario, 2020 diagnosis and treatment levels remained constant in subsequent years. In the Gradual Decline scenario, an annual decrease of 10% in both diagnoses and treatments was implemented, starting in 2020. WHO incidence target was disregarded, due to low HCV incidence in The Netherlands (≤5 per 100,000). Results: Following the Status Quo and Gradual Decline scenarios, The Netherlands would meet WHO’s elimination targets by 2027 and 2032, respectively. From 2015 to 2030, liver-related mortality would be reduced by 97% in the Status Quo and 93% in the Gradual Decline scenario. Compared to the Status Quo scenario, the Gradual Decline scenario would result in 12 excess cases of decompensated cirrhosis, 18 excess cases of hepatocellular carcinoma, and 20 excess cases of liver-related death from 2020–2030. Conclusions: The Netherlands is on track to reach HCV elimination by 2030. However, it is vital that HCV elimination remains high on the agenda to ensure adequate numbers of patients are being diagnosed and treated.

## 1. Introduction

Chronic viral hepatitis, if left untreated, leads to considerable morbidity and liver-related mortality [1]. Therefore, the World Health Organization (WHO) set ambitious hepatitis B (HBV) and C virus (HCV) elimination targets in 2016. The goal is to eliminate viral hepatitis as a public health threat by 2030, which is defined by the following targets: (1) 80% reduction in incidence, (2) 65% reduction in hepatitis-related mortality, (3) 90% diagnosis coverage, and (4) 80% treatment coverage [2]. The year 2015 serves as baseline for these targets. Many countries aim to reach these goals in time and elaborate efforts have been made to monitor progress towards elimination, often using mathematical models [3,4].

With regard to hepatitis C, it appears that only few countries are on track to meeting the WHO targets in time [5]. A recent modelling study, using the latest data on chronic HCV prevalence, and annual diagnosis and treatment levels in 45 high-income countries, suggests that only Australia, Canada, France, Germany, Iceland, Italy, Japan, Spain, Sweden, Switzerland, and the United Kingdom are currently on track [5]. Tailored HCV-specific national strategies, regional or national guidelines, national expert advisory groups and/or decentralized HCV screening likely keep these countries on a trajectory towards elimination.

The situation is different in The Netherlands. While there is a national plan that is endorsed by the Ministry of Health, the government has not allocated funds to aid its execution, and the plan itself lacks specific targets and accompanying interventions. Furthermore, The Netherlands does not yet have a nationwide hepatitis registry, complicating the ability to track our progress. However, physicians took the initiative to establish a national collaboration group (HepNed) to create the necessary infrastructure to eliminate HCV. HepNed has initiated several HCV elimination projects, such as CELINE and CAC.

CELINE, which stands for hepatitis C elimination in The Netherlands, is a nationwide retrieval project aiming to re-engage lost to follow-up HCV patients with care [6]. The project uses laboratory and patient records dating back 15 years from virtually all hepatitis treatment centers in The Netherlands. CAC, which stands for hepatitis C Chain of Addiction Care, is a project that aims to decentralize HCV care for people visiting addiction care services, one of the few remaining risk groups for chronic HCV infection in The Netherlands, even though transmission is very low [7]. Patients in several facilities all over The Netherlands are screened and linked to care, and data is collected throughout this process. These projects have provided us with high quality data on the current epidemiology of HCV in The Netherlands.

A recent study estimated that The Netherlands will reach the WHO HCV elimination targets by 2035 [5]. However, this study did not have access to the detailed epidemiologic data yielded from recent elimination projects. A previous Dutch modelling study from the pre-DAA era investigated various strategies to reduce the future HCV disease burden [8]. Many changes from their most effective strategy have since been implemented, including unrestricted access to direct-acting antivirals (DAA). Furthermore, various efforts to achieve viral hepatitis elimination have since been initiated. The aim of the present modelling study is therefore to evaluate the current timeline towards HCV elimination in The Netherlands.

## 2. Methods

### 2.1. The Model

We utilized a mathematical model developed by the Centre for Disease Analysis [4] to model the current progress towards HCV elimination as well as the effect of various interventions on HCV-associated outcomes. This model has been used extensively in various healthcare situations and countries [9,10,11,12,13,14]. Briefly, the Excel-based Markov model forecasts the future HCV-infected population and associated liver-related morbidity (decompensated cirrhosis and hepatocellular carcinoma) and mortality. The model uses an age- and gender-specific disease progression framework, previously detailed elsewhere [9]. It incorporates the WHO targets and forecasts when the country will reach these goals. Ethical approval from an institutional review board was not required for the execution of this study.

### 2.2. Model Base-Case Input

The model requires various parameters as base-case input (Table 1). These input parameters were based on the literature and/or consensus from expert meetings with HCV physicians and public health (modelling) experts from the National Institute for Public Health and the Environment and from Municipal Health Services, and are described in Table 1 and in detail below.

#### 2.2.1. Viraemic Prevalence

The prevalence of chronic HCV infection in The Netherlands in 2016 [16] was estimated by using the workbook method, originally developed to estimate the HIV/AIDS prevalence in low endemic countries with concentrated epidemics [18]. This study estimates that 22,885 people aged 15 years and older were ever chronically infected with HCV [16]. We adjusted this prevalence to include people aged 14 years or younger (Table 1), based on the age distribution detailed elsewhere [8].

The number of viraemic individuals in 2016 was calculated by subtracting the number of patients cured up to 2016 from the adjusted 2016 prevalence estimate. Treatment data were obtained from the GIP database, a web-based database from the Dutch National Health Care Institute that contains data on physician-prescribed medication in outpatient care [17]. Supplementary Table S1 displays (pegylated) interferon and DAA prescriptions from 2000–2016. These data reflect the annual total number of individual users, independent of treatment indication. As indications for (pegylated) interferon-based therapy expand beyond chronic HCV, we revised this data to reflect the treated and cured HCV population (Supplementary File S1 and Table S2). This resulted in an estimated population of 12,590 cured patients, leading to a baseline of 11,057 viraemic patients in 2016 (Table 1).

#### 2.2.2. HCV Incidence

The biggest influx of new HCV infections in The Netherlands is generated by first-generation migrants from HCV-endemic countries. An estimated 400 new chronic infections are introduced to The Netherlands yearly due to migration, based on annual migration statistics and published prevalence data [19,20]. The model incorporates these infections into the HCV incidence. True HCV incidence, due to active transmission, is estimated to be very low in The Netherlands. People who inject(ed) drugs (PWID) used to be a major HCV risk group in The Netherlands. However, due to the implementation of several successful harm reduction strategies, accompanied by a change in drug use culture, HCV incidence has declined [21]. After 2000, the primary risk group for HCV infection was no longer PWID, but men who have sex with men (MSM) [22,23]. Nowadays, almost all acute HCV cases occur among MSM [7]. The National Institute for Public Health and the Environment data from the previous 10 years show that, on average, the annual number of acute HCV cases is 54 (range 30–67) [7]. The incidence of HCV re-infection has increased over the last few years, with 26 re-infections reported in 2019 as compared to 2 in 2016 [24]. A recent study suggests that the WHO HCV incidence target may be hard to reach in countries where HCV incidence is already low [25]. The authors propose an adapted incidence goal: annual incidence ≤5 per 100,000 people. This adapted incidence goal has already been met, both in 2016 and 2019 [7,24]. We have therefore disregarded the WHO incidence goal incorporated in the model.

#### 2.2.3. Number of Diagnosed Individuals

Numbers of ever-diagnosed and annually diagnosed patients were based on CELINE project data (unpublished) [6]. Approximately 70% of ever-infected patients received a formal diagnosis, resulting in 3963 diagnosed but untreated people remaining at large in 2016 (Table 1). During 2016–2019, an average of 728 patients were newly diagnosed with viraemic HCV annually. This number corresponds with the number of 700 used in a similar modelling study by Hatzakis et al. [26].

#### 2.2.4. Number of Treated Individuals

Treatment data were obtained from the GIP database [17]. Data on HCV therapy and cure from 2000–2015 are presented in Supplementary File S1. Prior to 2016, DAA treatment was reserved for people with advanced disease (patients with F3 fibrosis or cirrhosis, liver transplant patients or candidates, and patients with severe extrahepatic manifestations). Since November 2015, all official restrictions on DAA treatment were lifted, resulting in widely available and reimbursed HCV treatment for everyone with health insurance. Therefore, SVR was assumed to be >95% during and after 2016. A total of 776 people were treated with DAAs in 2019 (see Supplementary Tables S2 and S3).

### 2.3. Model Scenarios

Our aim was to evaluate the Dutch timeline towards HCV elimination, starting in 2020. First, we intended to develop a scenario maintaining our elimination efforts on the same level as in 2019 (“Status Quo” scenario). As this might be an optimistic scenario, we also wanted to incorporate a scenario in which a yearly reduction in elimination efforts was implemented (“Gradual Decline” scenario). We also performed a sensitivity analysis, implementing a larger reduction in elimination efforts.

During the execution of this study, Coronavirus Disease 2019 (COVID-19) emerged, leading to a serious strain on healthcare in our country with devastating effects on non-COVID care [27,28]. Therefore, we implemented a substantial decrease in elimination efforts in both scenarios. This decrease was implemented for two years, as a one-year delay was deemed too optimistic. This two-year delay in the Status Quo scenario resulted in the Two-year COVID-19 Delay scenario, whereas the delay in the Gradual Decline scenario resulted in the Post-recovery Gradual Decline Scenario. All scenarios are detailed below.

#### 2.3.1. Status Quo Scenario

The annual number of treated patients peaked in 2015, just after the introduction of DAAs, but declined continuously thereafter (Supplementary Figure S1). For the Status Quo scenario, we assumed that this decline would reach its plateau in 2020. We therefore reduced the number of annual treatments with 10% as compared to 2019, and applied a similar reduction to the annual number of diagnosed patients. From 2021 onwards, these numbers were modelled to remain equal to 2020. The scenario inputs can be found in Supplementary Table S4.

#### 2.3.2. Gradual Decline Scenario

In the second scenario (“Gradual Decline”), we assumed a continuous reduction of 10% per year in both the number of annual newly diagnosed and treated patients, starting in 2021. The Gradual Decline scenario model inputs can be found in Supplementary Table S5. Furthermore, a sensitivity analysis was run on this scenario, to assess the impact of a larger reduction in elimination efforts (“Sensitivity Analysis”). An annual reduction of 15% in newly diagnosed and treated patients was therefore implemented, starting in 2021. Other scenario variables were not altered. The Sensitivity Analysis model inputs can be found in Supplementary Table S6.

#### 2.3.3. COVID-19 Scenarios

A recent study from the United States investigated the impact of the COVID-19 pandemic on HCV care by comparing the number of newly diagnosed patients during a three-month-period before COVID-19 measures with the subsequent three months. The authors found a 42% reduction in the number of new diagnoses [29]. To model the impact of COVID-19 on HCV elimination in The Netherlands, we assumed a similar decrease in diagnosis levels and furthermore assumed that the same decrease would also apply to the number of annually treated patients. In the third scenario (Two-year COVID-19 Delay), these reductions were assumed for 2020 and 2021, and model parameters were assumed to return to Status Quo values in 2022 and remain stable thereafter. The fourth scenario (Post-COVID Recovery Gradual Decline) assumed the same two-year delay in 2020–2021 and initial recovery in 2022, but furthermore assumed a continuous annual reduction of 10% in both newly diagnosed and treated patients from 2023 onwards. All model inputs for COVID-related scenarios can be found in Supplementary Tables S7 and S8.

## 3. Results

An estimated 11,327 patients were HCV-infected in 2016, of whom 3963 were estimated to be diagnosed. Following the Status Quo scenario of 630 new diagnoses and 698 treated patients annually, the WHO targets would be met by 2027 (Table 2). The incidence target, which was disregarded due to the extremely low pre-existing incidence in The Netherlands, would be met in 2034. In the Gradual Decline scenario, in which a yearly 10% reduction in diagnoses and treatments was implemented, WHO elimination targets would be met by 2032. The incidence target would not be met. All COVID-19-related scenario outcomes are detailed in Supplementary File S2, Figures S2 and S3, and Table S9. In general, an estimated 360 patients need to be treated annually from 2020–2030 in order to meet the treatment target by 2030.

**Figure 1:**
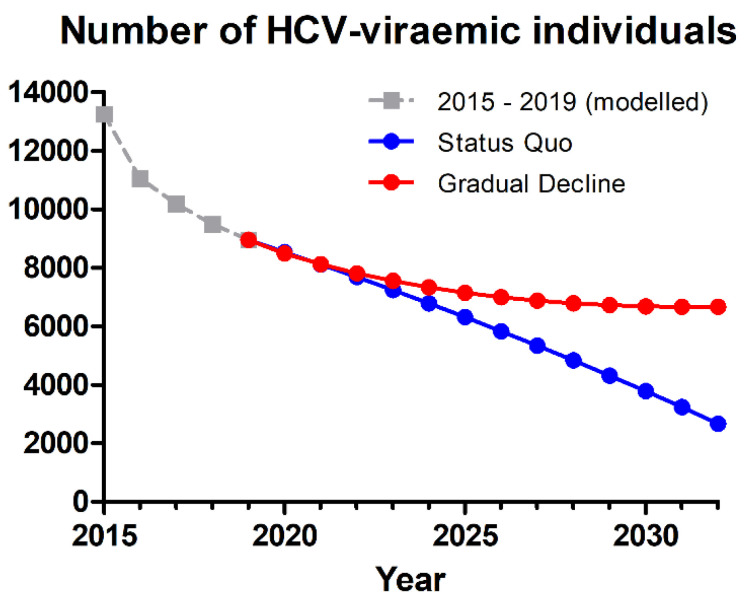
Predicted number of HCV-viraemic individuals in The Netherlands over time, following the Status Quo and Gradual Decline scenarios. HCV: hepatitis C virus.

**Figure 2:**
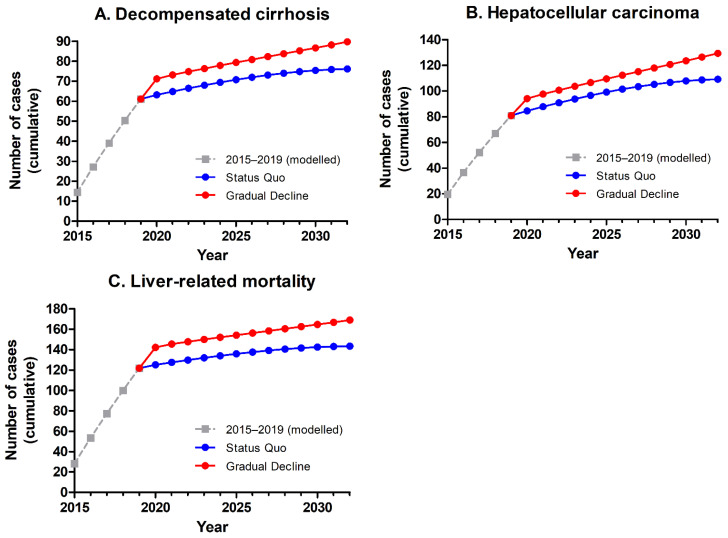
Predicted incident cases (cumulative) of (**A**) decompensated cirrhosis, (**B**) hepatocellular carcinoma, and (**C**) liver-related mortality in The Netherlands over time, following the Status Quo and Gradual Decline scenarios.

All scenarios had a significant impact on the number of viraemic people (see Figure 1). The Status Quo scenario reduced viraemic HCV prevalence by 71% from 2015 to 2030, while the corresponding reduction in the Gradual Decline scenario was 50%. During the same time period, liver-related mortality was reduced by 97% in the Status Quo and 93% in the Gradual Decline scenario. Outcomes regarding liver-related morbidity and mortality are shown in Figure 2. The Gradual Decline scenario resulted in 12 excess cases of decompensated cirrhosis, 18 excess cases of hepatocellular carcinoma (HCC), and 20 excess cases of liver-related death from 2020–2030, compared to the Status Quo scenario.

The sensitivity analysis showed that a 15% reduction in annual diagnoses and treatments, as opposed to the 10% implemented in the Gradual Decline scenario, pushed back the WHO elimination targets significantly (see Table 3). The incidence target was not met, comparable to the Gradual Decline scenario. Furthermore, after an initial decrease, HCV prevalence started increasing from 2028 onward. The difference in liver-related morbidity and mortality was small, with one excess case of decompensated cirrhosis, two excess cases of hepatocellular carcinoma, and one excess case of liver-related death from 2020–2030, compared to the Gradual Decline scenario.

## 4. Discussion

The aim of this study was to predict when The Netherlands will meet the WHO HCV elimination targets. The results show that The Netherlands is on track to eliminate hepatitis C by 2030, if annual diagnosis and treatment rates can be maintained at 2019 levels. When an annual decrease of 10% was implemented for both diagnosis and treatment levels from 2021 onwards, WHO elimination targets were met by 2032. Both scenarios had a significant impact on viraemic prevalence and liver-related morbidity and mortality. Interestingly, the absolute numbers of incident cases of decompensated cirrhosis, hepatocellular carcinoma, and liver-related mortality sharply dropped, starting in 2020. This might be explained by the history of the HCV epidemic in The Netherlands.

The HCV epidemic took off during the heroin crisis in the 1970s, resulting in a wave of HIV and HCV infections [21]. Injecting drug use continuously decreased from 1985 to 2015, and concordantly, HIV and HCV incidence also dropped [21]. After 2000, a shift in HCV incidence from PWID to MSM was seen [22,23]. HCV infection is likely detected early in MSM due to regular testing, and treatment uptake in this group is high [30]. HCV-related morbidity and mortality in diagnosed MSM is therefore low. As most PWID have been infected from 1970–1990, the resulting peak in morbidity and mortality has most likely passed. When DAAs became available in 2014–2015, treatment was only reserved for people with F3 or F4 fibrosis. Combined with the continuous use of DAA therapy for all patients over the next few years, this may have resulted in a sharp decline in liver-related morbidity and mortality, as shown by our results. However, these modelled results need to be validated using real-life data. Hopefully, the future national HCV registry, currently in its pilot phase, will provide accurate data on HCV-related epidemiology, morbidity, and mortality.

Our results are more favourable than those of a recent study which estimated that The Netherlands would meet HCV elimination targets by 2035 [5]. The authors concluded that both the 90% diagnosis coverage and the 80% treatment coverage would be the first targets to be met, in 2025, and that the 65% reduction in liver-related mortality would follow in 2035. Remarkably, our study contrasts with these results, which may have various explanations. First, the base case prevalence used in our study differed from previously published studies using this model. In the current study, we estimated the number of currently viraemic people by subtracting the number of cured patients from the ever-infected population, using a high-quality treatment database and the most recent prevalence estimate [16,17]. This led to a slightly lower base-case viraemic prevalence compared to other studies. Furthermore, due to the larger number of cured patients, it is likely that morbidity and mortality outcomes appeared more favourable compared to other studies that used different methods. A third reason, which explains the difference regarding the treatment target, is the timing of the performed studies. As shown in Supplementary Figure S1, treatment numbers peaked after the introduction of DAAs (2015–2016) but declined shortly thereafter (2017–2019). It is possible that other, earlier studies extrapolated treatment numbers from the “peak” period, leading to an overestimation of subsequent treatment levels.

In view of the current pandemic, we modelled two scenarios projecting the impact of COVID-19. Both scenarios assumed a 42% reduction to Status Quo 2020 levels of annual diagnoses and treatments for two years, recovering to the Status Quo 2020 level in 2022. This reduction was based on a recent study from the United States [29], as Dutch data at the time of execution of this study was lacking. However, a recently published study showed that Dutch HCV diagnoses in 2020 decreased by 43% as compared to 2019, and that the weekly relative reduction mirrored the weekly number of COVID-19 admissions [31]. Furthermore, recently published treatment data by the GIP database show that 505 people have been treated for HCV in 2020, corresponding to a 35% decrease as compared to 2019 [17]. These data support the robustness of the COVID-19 scenario inputs. In the first COVID-19 scenario, diagnosis and treatment rates were kept constant after initial recovery in 2022, whereas the second assumed a 10% annual reduction from 2023 onwards. Remarkably, both scenarios resulted in earlier elimination than the Gradual Decline scenario, mainly due to the 90% diagnosis coverage target. This can be explained by the higher absolute number of new diagnoses and treatments during 2020–2030 in both COVID-19 scenarios compared to the Gradual Decline scenario. However, the number of liver-related deaths is higher for the COVID-19 scenarios (17 and 19 additional deaths, respectively, compared to the Gradual Decline scenario), which is also reflected in the year in which the 65% reduction in liver-related mortality is reached (2022 in both COVID-19 scenarios, compared to 2021 in the Gradual Decline scenario). Furthermore, both COVID-19 scenarios resulted in more cases of decompensated cirrhosis and hepatocellular carcinoma, although absolute numbers remain small.

The sensitivity analysis emphasizes the lack of flexibility in maintaining annual diagnosis and treatment levels in a low-prevalence country such as The Netherlands. A 15% reduction in these levels, as opposed to the 10% reduction in the Gradual Decline scenario, immediately resulted in the diagnosis target becoming unattainable before 2050. A 20% reduction resulted in the treatment target to be unattainable as well (results not shown). Eventually, the sensitivity analysis even resulted in an increase in viraemic HCV prevalence. This analysis therefore emphasizes the need to maintain high diagnosis and treatment levels in the upcoming years. However, maintaining high diagnosis and treatment levels may prove challenging. Unpublished data from the nationwide retrieval project (CELINE) on annual new diagnoses show a continuous decrease in the number of new diagnoses over the last five years, and GIP database data on annually treated patients show a similar decrease. Groups in The Netherlands with the highest absolute number of (prior) chronic HCV infections are first-generation migrants from endemic countries, PWID, and people who have no (identified) risk factor for HCV infection [16]. These groups are harder to reach compared to other HCV risk groups. Fortunately, there are stakeholders in The Netherlands that aim to improve HCV care for these groups. Migrant screening, decentralization of HCV care in addiction care (CAC), and screening of prisoners are items currently high on the agenda. These efforts are vital in order to eliminate hepatitis C as a public health threat in The Netherlands. However, more support from the government is needed to enable these efforts.

## 5. Strengths and Limitations

This is the first Dutch modelling study that estimates the timing of the WHO elimination targets. We incorporated the most recent, published data, as well as unpublished data that has been collected during an ongoing nationwide retrieval project (CELINE). This unpublished data has confirmed previously published data, supported expert opinion, and given new insights into the Dutch HCV epidemic, strengthening the current analysis. Four realistic scenarios were devised, resulting in a robust elimination timeline. However, this study also has several limitations.

The model is limited by the accuracy of its input parameters. Unfortunately, as country-specific data was often missing, certain assumptions had to be made. In addition, the model itself makes certain assumptions as well. The annual number of HCV drug users was approximated based on GIP database data, which incorporated various assumptions, especially for the pre-DAA era. It is possible that people have been counted more than once, due to timing of treatment, treatment duration, and possible re-treatment after initial failure or re-infection. Furthermore, the model assumes that the distribution of treatments runs concordant to the genotype distribution and is equal in all risk groups. In reality, some genotypes and/or key populations were less likely to be treated due to suboptimal treatment results or barriers to treatment. Lastly, the model does not account for different SVR percentages after re-treatment due to failure or re-infection. These assumptions may have resulted in an overestimation of the number of treated and thereby cured patients, resulting in an underestimation of viraemic prevalence. Hopefully, once the national HCV registry is established, more accurate data on epidemiology, treatment, and (long-term) clinical outcomes will be available.

## 6. Conclusions

In conclusion, The Netherlands appears to be on track to reach HCV elimination by 2030, though many challenges remain. This study demonstrates what it takes to meet the elimination targets in time, which might guide us in developing and implementing the (public) health policies that are needed. Dutch HCV elimination still needs invested stakeholders to maintain and, where necessary, improve the existing infrastructures regarding HCV care. These study results should be used as a base with which we can compare our actions in the future.

## Figures and Tables

**Table 1:** Base Case Model Inputs.

Variable	Input	Source
Size of overall population (2016)	16,890,864	United Nations [15]
Ever-infected patients with chronic HCV (up to 2016)	23,647	2016 prevalence [16], adjusted to include people < 15 years old
Total number of viraemic patients (2016)	11,057	Based on the adjusted 2016 prevalence [16] and the estimated number of cured patients up to 2016
Ever-diagnosed patients (up to 2016)	16,533	CELINE data (unpublished)
Total number of diagnosed patients (2016)	3963	Based on CELINE data and the estimated number of cured patients up to 2016
Number of annual newly diagnosed patients (2016)	700	CELINE data (unpublished)
Number of annual treated patients		GIP database [17]
2016	2647	
2017	1173	
2018	988	
2019	776	
Fibrosis stage restriction (2016)	≥F0	No treatment restrictions since 2016
Maximum age eligible for treatment (2016)	85+	No treatment restrictions since 2016
Average SVR (2016)	95%	See Supplementary File S1

**Table 2:** Forecasted year of elimination with scenarios “status quo” and “gradual decline”.

WHO’s Elimination Target	Year of Elimination	
	Status Quo	Gradual Decline
65% reduction in liver-related mortality	2020	2021
90% of infected patients diagnosed	2027	2032
80% of eligible patients treated	2025	2027
**Year of elimination**	2027	2032

**Table 3:** Forecasted year of elimination in the sensitivity analysis.

WHO’s Elimination Target	Year of Elimination
65% reduction in liver-related mortality	2021
90% of infected patients diagnosed	>2050
80% of eligible patients treated	2030
**Year of elimination**	>2050

## Data Availability

The data presented in this study are available on request from the corresponding author.

## Supplementary Materials

The following are available online at https://www.mdpi.com/article/10.3390/jcm10194562/s1, File S1: Available treatments and SVR percentages in The Netherlands, File S2: COVID-19 scenario results, Table S1: Total number of annual HCV antiviral drug users in The Netherlands, Table S2: Approximation of the number of annual HCV antiviral drug users for HCV infection in The Netherlands, Table S3: Calculated genotype-dependant SVR percentages during the (pegylated) interferon era (2000–2014), Table S4: Status Quo scenario model inputs, Table S5: Gradual decline scenario model inputs, Table S6: Sensitivity analysis model inputs, Table S7: Two-year COVID-19 Delay model inputs, Table S8: Post-COVID Recovery Gradual Decline model inputs, Table S9: Forecasted year of elimination with scenarios “Two-year COVID-19 Delay” and “Post-COVID Recovery Gradual Decline”, Figure S1: Actual (continuous line) and predicted (dotted lines) number of patients treated with direct acting antivirals, Figure S2: Predicted number of HCV-viraemic individuals in The Netherlands over time, following the Two-year COVID-19 Delay and Post-recovery Gradual Decline scenarios, Figure S3: Predicted incident cases (cumulative) of (A) decompensated cirrhosis, (B) hepatocellular carcinoma, and (C) liver-related mortality in The Netherlands over time, following the Two-year COVID-19 Delay and Post-recovery Gradual Decline scenarios.
