# Supplementary material for: The Netherlands Is on Track to Meet the World Health Organization Hepatitis C Elimination Targets by 2030
Source: J Clin Med. 2021 Sep 30;10(19):4562. doi: 10.3390/jcm10194562 (PMC8509638; doi:10.3390/jcm10194562)
Supplement: Supplementary file 1 [file jcm-10-04562-s001.zip › jcm-1368881-supplementary.pdf]

Supplementary Data Index

Supplementary File S1 2

Supplementary Table S1 4

Supplementary Table S2 5

Supplementary Table S3 5

Supplementary Figure S1 8

Supplementary Table S4 6

Supplementary Table S5 6

Supplementary Table S6 7

Supplementary Table S7 7

Supplementary Table S8 7

Supplementary File S2 7

Supplementary Table S9 7

Supplementary Figure S2 8

Supplementary Figure S3 9

References 10

Supplementary File S1: Available treatments and SVR percentages in the Netherlands

Interferon alfa 2a and 2b

In 2003, both pegylated interferon alfa 2b and 2a were available in the Netherlands. We therefore assumed that from 2004 onwards, interferon alfa 2a and 2b were not prescribed for HCV patients anymore. We therefore excluded interferon treatments after 2003. However, treatments for other indications had to be subtracted from data from 2000-2003. Therefore, we calculated the average number of patients who were annually treated with interferon 2a and 2b from 2004-2019. Subsequently, we subtracted these numbers from the annual number of treated patients in 2000-2003, resulting in numbers of patients only treated for HCV (see Supplementary Table S2).

Peginterferon alfa 2b

Peginterferon alfa 2b is only registered for HCV, since 2001. We assumed that from 2015 onwards, HCV was no longer treated with pegylated interferon 2b. However, there were still some annual users. The other peginterferon, pegylated interferon alfa 2a, is registered for HBV since 2005. We hypothesized that since 2005, pegylated interferon alfa 2b has also been used for HBV. It is also possible that peginterferon alfa 2b has been used for a different off-label indication, that we are currently unaware of. We calculated the average number of patients annually treated from 2015-2019 and subtracted this number from the annual number of users from 2005-2014, resulting in the number of patients treated for HCV. Treatment data from 2001-2004 were assumed to be solely attributable to HCV (see Supplementary Table S2).

Peginterferon alfa 2a

Peginterferon alfa 2a is registered for HCV since 2003. Since 2005, it is also registered for HBV. Treatment data from 2003 and 2004 are therefore solely attributable to HCV. We assumed that from 2015 onwards, HCV was no longer treated with pegylated interferon 2a. However, high treatment numbers remain, since peginterferon alfa 2a has in recent years also been prescribed off-label for polycythaemia vera. We assumed that the number of patients treated for HBV with peginterferon alfa 2a were similar to those treated with peginterferon alfa 2b. Therefore, we subtracted the average number of annually treated patients with peginterferon alfa 2b in 2015-2019 from the number of annually treated patients with peginterferon alfa 2a in 2005-2014 (see Supplementary Table S1 & S2).

Telaprevir and boceprevir

Telaprevir and boceprevir were available since 2012 and were prescribed with peginterferon and ribavirin to treat genotype 1 infected patients. Since there was no other indication for triple therapy, treatment data from 2012-2015 were assumed to be solely attributable to HCV (see Supplementary Table S2).

Other DAA regimens

Sofosbuvir has been available in the Netherlands since November 2014 and could be prescribed with peginterferon and/or ribavirin. In 2014, only 77 patients have been treated with sofosbuvir. The role of sofosbuvir on the total number of cured patients in 2014 was assumed to be insignificant. Since 2015, this drug could also be prescribed in combination with daclatasvir or simeprevir. As mentioned previously, we assumed that since 2015, no patients were treated with a peginterferon-containing regimen. Therefore, when calculating the annual number of DAA-treated patients, we only counted sofosbuvir, since simeprevir and daclatasvir had to be prescribed in combination with sofosbuvir. For the same reason only ombitasvir/paritaprevir/ritonavir users were counted, since dasabuvir could only be prescribed in combination with this drug. See Supplementary Table S2 for the resulting annual DAA users.

Genotype-dependent SVR percentages

SVR percentages were calculated in a genotype-dependent manner. Genotype 1 is the most prevalent genotype in the Netherlands (50%), followed by genotype 3 (30%) and genotype 2 and 4 (both 10%) [1]. We assumed that all (peg)interferon-treated patients were treated in conjunction with ribavirin. Average genotype-dependant SVR percentages for the (pegylated) interferon era (2000 – 2014) were calculated based on the genotype distribution and literature-based SVR percentages (see Supplementary Table S3) [2-4]. SVR percentages for DAAs were also literature-based, but genotype-independent. Sofosbuvir has been available in the Netherlands since November 2014 and was at first only reimbursed for patients with F3 fibrosis or cirrhosis, patients who underwent or were waiting for liver transplantation and patients with severe extrahepatic manifestations. This changed in November 2015, when sofosbuvir was reimbursed for all patients. Due to these restrictions, it was assumed that a relatively higher proportion of cirrhotic patients was treated in 2015. Therefore, an SVR of 90% was used for 2015, compared to an SVR of >95% from 2016 onwards.

Supplementary File S2: COVID-19 scenario results

In the Two-year COVID-19 Delay scenario, elimination was pushed back by one year, compared to the Status Quo scenario (Supplementary Table S9). When an annual reduction of 10% was implemented in both newly diagnosed and treated patients after initial recovery (Post-COVID-19 Recovery Gradual Decline scenario), elimination targets were met by 2030.

Both COVID-19 scenarios had a significant impact on the number of viraemic people (see Supplementary Figure S2). The Two-year COVID-19 Delay scenario reduced viraemic HCV prevalence by 67% from 2015 to 2030, while the prevalence reduction in the Post-recovery Gradual Decline scenario was 52%. During the same time period, liver-related mortality was reduced by 96% in the Two-year COVID-19 Delay and 93% in the Post-recovery Gradual Decline scenarios. Outcomes regarding liver-related morbidity and mortality are shown in Supplementary Figure S3. The COVID-19-related scenarios both had a larger impact on morbidity and mortality than the Gradual Decline scenario. The Two-year COVID-19 Delay and Post-recovery Gradual Decline scenarios resulted in respectively 6 and 8 cases of decompensated cirrhosis, 5 and 6 cases of HCC, and 17 and 19 cases of liver-related death. Compared to the Status Quo scenario, there were 18 and 20 excess cases of decompensated cirrhosis, 23 and 26 excess cases of HCC, and 37 and 39 excess cases of liver-related death during the same time period.

Supplementary Table S1

Table S1: Total number of annual HCV antiviral drug users in The Netherlands.

Therapy	2000	2001	2002	2003	2004	2005	2006	2007	2008	2009	2010	2011	2012	2013	2014	2015	2016	2017	2018	2019
**Interferon alfa 2a**	604	463	349	327	315	320	347	202	147	164	164	149	123	106	91	92	74	80	82	83
**Interferon alfa 2b**	1196	1021	760	611	393	247	225	198	188	165	123	93	88	70	75	69	52	41	37	8
**Peginterferon alfa 2b**	19	415	879	1023	877	814	678	662	676	502	424	363	386	367	229	177	148	136	7	-
**Peginterferon alfa 2a**	-	-	-	144	436	705	795	869	897	934	980	974	1132	991	607	503	463	614	694	837
**Telaprevir**	-	-	-	-	-	-	-	-	-	-	-	-	207	177	42	1	-	-	-	-
**Boceprevir**	-	-	-	-	-	-	-	-	-	-	-	-	198	194	46	17	-	-	-	-
**Simeprevir**	-	-	-	-	-	-	-	-	-	-	-	-	-	-	-	533	77	13	1	-
**Daclatasvir**	-	-	-	-	-	-	-	-	-	-	-	-	-	-	-	660	951	268	37	2
**Sofosbuvir**	-	-	-	-	-	-	-	-	-	-	-	-	-	-	77	1359	1076	292	59	8
**Dasabuvir**	-	-	-	-	-	-	-	-	-	-	-	-	-	-	-	84	149	21	2	-
**Sofosbuvir/Ledipasvir**	-	-	-	-	-	-	-	-	-	-	-	-	-	-	-	323	1395	524	197	40
**Ombitasvir/Paritaprevir/ Ritonavir**	-	-	-	-	-	-	-	-	-	-	-	-	-	-	-	102	176	25	3	-
**Elbasvir/Grazoprevir**	-	-	-	-	-	-	-	-	-	-	-	-	-	-	-	-	-	58	43	96
**Sofosbuvir/Velpatasvir**	-	-	-	-	-	-	-	-	-	-	-	-	-	-	-	-	-	274	357	218
**Sofosbuvir/Velpatasvir/ Voxilaprevir**	-	-	-	-	-	-	-	-	-	-	-	-	-	-	-	-	-	-	11	20
**Glecaprevir/Pibrentasvir**	-	-	-	-	-	-	-	-	-	-	-	-	-	-	-	-	-	-	318	394

Supplementary Table S2

Table S2: Approximation of the number of annual HCV antiviral drug users for HCV infection in The Netherlands.

Therapy	2000	2001	2002	2003	2004	2005	2006	2007	2008	2009	2010	2011	2012	2013	2014	2015	2016	2017	2018	2019	TOTAL
**Interferon alfa 2a**	445	304	190	168	-	-	-	-	-	-	-	-	-	-	-	-	-	-	-	-	1107
**Interferon alfa 2b**	1066	891	630	481	-	-	-	-	-	-	-	-	-	-	-	-	-	-	-	-	3068
**Peginterferon alfa 2b**	-	415	879	1023	877	697	561	545	559	385	307	246	269	250	112	-	-	-	-	-	7125
**Peginterferon alfa 2a**	-	-	-	144	436	588	678	752	780	817	863	857	1015	874	490	-	-	-	-	-	8294
**(Peg)interferon-free DAA therapy**	-	-	-	-	-	-	-	-	-	-	-	-	-	-	-	1784	2647	1173	988	776	7368
**Total number of annual users**	1511	1610	1699	1816	1313	1285	1239	1297	1339	1202	1170	1103	1284	1124	602	1784	2647	1173	988	776	26962

Supplementary Table S3

Table S3: Calculated genotype-dependant SVR percentages during the (pegylated) interferon era (2000 – 2014).

Genotype	Pre-triple therapy era (2000 – 2011)	Triple therapy era (2012 – 2014)
Genotype 1	40,8%	69,9%
Genotype 2/3	74,3%	74,3%
Genotype 4	41,8%	41,8%

Supplementary Table S4

Table S4: Status Quo scenario model inputs.

**Variable**													
Number of	**2020**	**2021**	**2022**	**2023**	**2024**	**2025**	**2026**	**2027**	**2028**	**2029**	**2030**	**2031**	**2032**
• Newly diagnosed patients	630	630	630	630	630	630	630	630	630	630	630	630	630
• Treated patients	698	698	698	698	698	698	698	698	698	698	698	698	698
Fibrosis stage restriction	>=F0												
Maximum age eligible for treatment	85+												
Average SVR	95%												

Supplementary Table S5

table S5: Gradual decline scenario model inputs.

**Variable**													
Number of	**2020**	**2021**	**2022**	**2023**	**2024**	**2025**	**2026**	**2027**	**2028**	**2029**	**2030**	**2031**	**2032**
• Newly diagnosed patients	630	567	510	459	413	372	335	301	271	244	220	198	178
• Treated patients	698	628	565	508	457	411	370	333	300	270	243	219	197
Fibrosis stage restriction	>=F0												
Maximum age eligible for treatment	85+												
Average SVR	95%												

Supplementary Table S6

Table S6: Sensitivity analysis model inputs.

Variable													
Number of	**2020**	**2021**	**2022**	**2023**	**2024**	**2025**	**2026**	**2027**	**2028**	**2029**	**2030**	**2031**	**2032**
• Newly diagnosed patients	630	536	455	387	329	280	238	202	172	146	124	105	90
• Treated patients	698	593	504	429	364	310	263	224	190	162	137	117	99
Fibrosis stage restriction	>=F0												
Maximum age eligible for treatment	85+												
Average SVR	95%												

Supplementary Table S7

Table S7: Two-Year COVID-19 delay model inputs.

Variable													
Number of	**2020**	**2021**	**2022**	**2023**	**2024**	**2025**	**2026**	**2027**	**2028**	**2029**	**2030**	**2031**	**2032**
• Newly diagnosed patients	365	365	630	630	630	630	630	630	630	630	630	630	630
• Treated patients	405	405	698	698	698	698	698	698	698	698	698	698	698
Fibrosis stage restriction	>=F0												
Maximum age eligible for treatment	85+												
Average SVR	95%												

Supplementary Table S8

Table S8: Post-COVID Recovery Gradual Decline model inputs.

Variable													
Number of	**2020**	**2021**	**2022**	**2023**	**2024**	**2025**	**2026**	**2027**	**2028**	**2029**	**2030**	**2031**	**2032**
• Newly diagnosed patients	365	365	630	567	510	459	413	372	335	301	271	244	220
• Treated patients	405	405	698	628	565	508	457	411	370	333	300	270	243
Fibrosis stage restriction	>=F0												
Maximum age eligible for treatment	85+												
Average SVR	95%												

Supplementary Table S9

Table S9. Forecasted year of elimination with scenarios "Two-year COVID-19 Delay" and "Post-COVID Recovery Gradual Decline".

WHO's elimination target	Year of elimination	
	Two-year COVID-19 Delay	Post-COVID Recovery Gradual Decline
80% reduction in HCV incidence (disregarded due to extremely low incidence in the Netherlands)	2035	-
65% reduction in liver-related mortality	2022	2022
90% of infected patients diagnosed	2028	2030

80% of eligible patients treated	2026	2027
**Year of elimination**	2028	2030

Supplementary Figure S1

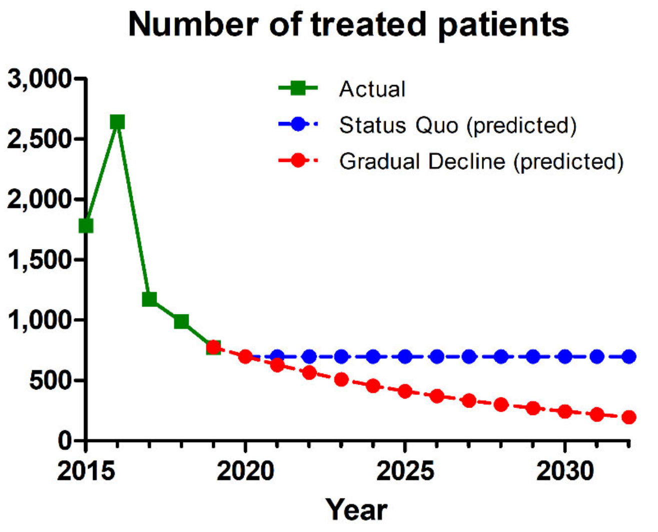

Figure S1. Actual (continuous line) and predicted (dotted lines) number of patients treated with direct acting antivirals.

Supplementary Figure S2

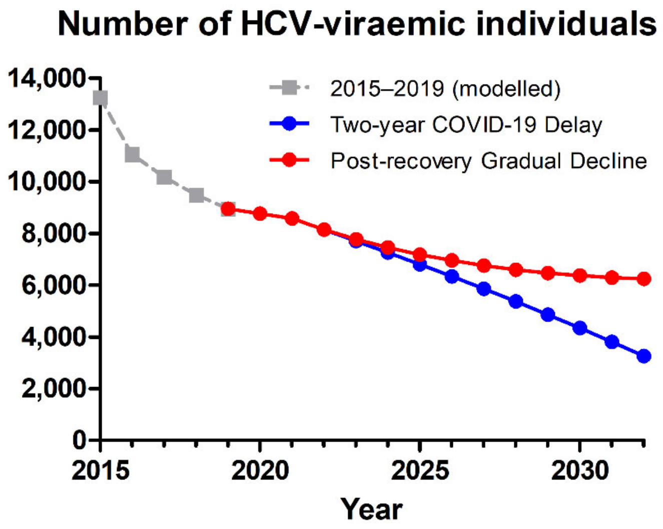

Figure S2. Predicted number of HCV-viraemic individuals in the Netherlands over time, following the Two-year COVID-19 Delay and Post-recovery Gradual Decline scenarios. HCV: hepatitis C virus; COVID-19: Coronavirus Disease 2019.

Supplementary Figure S3

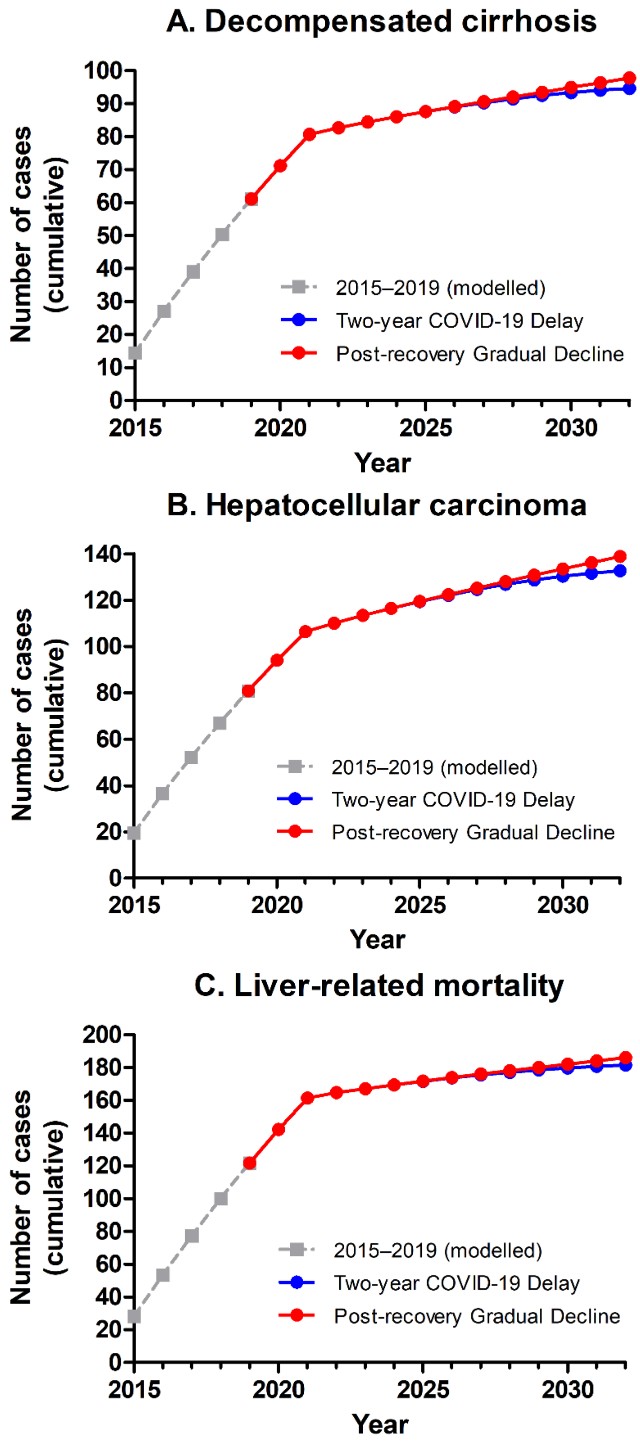

Figure S3. Predicted incident cases (cumulative) of (**A**) decompensated cirrhosis, (**B**) hepatocellular carcinoma, and (**C**) liver-related mortality in the Netherlands over time, following the Two-year COVID-19 Delay and Post-recovery Gradual Decline scenarios. COVID-19: Coronavirus Disease 2019.

References

1. de Vries, M.J.; te Rijdt, B.; van Nieuwkerk, C.M. Genotype distribution amongst hepatitis C patients in The Netherlands. *Neth. J. Med*. **2006**, *64*, 109–13.
2. Hauser, G.; Awad, T.; Brok, J.; Thorlund, K.; Štimac, D.; Mabrouk, M.; Gluud, C.; Gluud, L.L. Peginterferon plus ribavirin versus interferon plus ribavirin for chronic hepatitis C. *Cochrane Database Syst. Rev*. **2014**, *2*, Cd005441.
3. Hauser, G.; Awad, T.; Thorlund, K.; Štimac, D.; Mabrouk, M.; Gluud, C. Peginterferon alpha-2a versus peginterferon alpha-2b for chronic hepatitis C. *Cochrane Database Syst. Rev*. **2014**, *2*, Cd005642.
4. Kieran, J.; Schmitz, S.; O'Leary, A.; Walsh, C.; Bergin, C.; Norris, S.; Barry, M. The relative efficacy of boceprevir and telaprevir in the treatment of hepatitis C virus genotype 1. *Clin. Infect. Dis.* **2013**, *56*, 228–35.
